# Regional Differentiation of Retinoic Acid-Induced Human Pluripotent Embryonic Carcinoma Stem Cell Neurons

**DOI:** 10.1371/journal.pone.0016174

**Published:** 2011-01-20

**Authors:** Dennis E. Coyle, Jie Li, Mark Baccei

**Affiliations:** Department of Anesthesiology, University of Cincinnati, Cincinnati, Ohio, United States of America; University of Southern California, United States of America

## Abstract

The NTERA2 cl D1 (NT2) cell line, derived from human teratocarcinoma, exhibits similar properties as embryonic stem (ES) cells or very early neuroepitheial progenitors. NT2 cells can be induced to become postmitotic central nervous system neurons (NT2N) with retinoic acid. Although neurons derived from pluripotent cells, such as NT2N, have been characterized for their neurotransmitter phenotypes, their potential suitability as a donor source for neural transplantation also depends on their ability to respond to localized environmental cues from a specific region of the CNS. Therefore, our study aimed to characterize the regional transcription factors that define the rostocaudal and dorsoventral identity of NT2N derived from a monolayer differentiation paradigm using quantitative PCR (qPCR). Purified NT2N mainly expressed both GABAergic and glutamatergic phenotypes and were electrically active but did not form functional synapses. The presence of immature astrocytes and possible radial glial cells was noted. The NT2N expressed a regional transcription factor code consistent with forebrain, hindbrain and spinal cord neural progenitors but showed minimal expression of midbrain phenotypes. In the dorsoventral plane NT2N expressed both dorsal and ventral neural progenitors. Of major interest was that even under the influence of retinoic acid, a known caudalization factor, the NT2N population maintained a rostral phenotype subpopulation which expressed cortical regional transcription factors. It is proposed that understanding the regional differentiation bias of neurons derived from pluripotent stem cells will facilitate their successful integration into existing neuronal networks within the CNS.

## Introduction

The field of human embryonic stem (hES) cell research, although young, holds a potential for advancing our understanding of the mechanisms and pathways of human development and may provide an unlimited source of human neuronal cells for basic and applied research, therapeutic drug testing, and tissue repair or replacement. Yet our current understanding and use of hES cells for differentiation into neuron and glial subtypes is limited by the legal, ethical, and political considerations imposed on the use of these cells as well as the technical aspects of culturing these cells.

Another source of human pluripotent cells is embryonic carcinoma (EC) cells derived from human male germ cell tumors. Human EC (hEC) cells resemble hES cells in antigen expression patterns, developmental potential, and global gene expression including common gene expression patterns for both “stemness” and pluripotency [1], [2], [3], [4], [5], [6]. Unlike hES cells, most hEC cells are simple to passage, do not require feeder support for propagation and do not undergo spontaneous differentiation. The NTERA-2 cl. D1 (NT2) cell line is one of the characterized hEC cell lines used as a model system for differentiation of cells from the neural lineage [for review see [7]]. The NT2 neurons (NT2N) derived from NT2 cells are post-mitotic polarized cells that express neurofilaments, generate action potentials and calcium spikes, express, release, and respond to neurotransmitters. They have been used as cell grafts for a rat stroke model where they mature and display functional integration as well as in phase 1 and 2 clinical trials with stable stroke patients [2], [8], [9], [10], [11], [12], [13], [14], [15]. Studies have also reported that NT2 cells are capable of differentiating into astrocytes [16], [17]. Recently, NT2 cells have been reported to be capable of generating all three germ layers when differentiation was induced by *in vitro* generation of embryoid bodies [5].

Neural progenitors carry their positional identity which on further differentiation defines the phenotype and activity of the neurons generated. This positional identity makes these progenitors more responsive to environmental cues for a specific neural region which is of critical importance in transplantation. Although the type of neurotransmitters expressed by NT2N was first investigated by Guillemain et al. the lack of spatial identity information results in the inability to determine (for example) if a tyrosine hydroxylase expressing catecholaminergic neuron is of midbrain, forebrain or hypothalamus fate [2]. To address this knowledge gap, we performed quantitative gene expression analysis of both NT2 cells and purified NT2N to quantify the regulation of the expression of transcription factors which define the regional identities of neural progenitors and neural neurotransmitter phenotypes following retinoic acid differentiation.

## Results

### Differentiation of NT2 cells yields neuronal and glial populations

**Figure 1:**
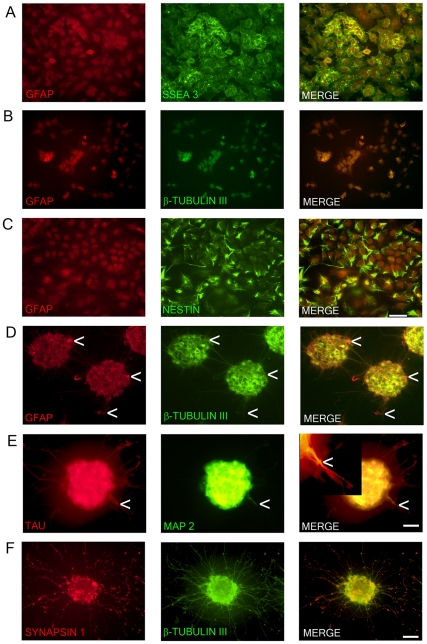
Marker expression of NTERA2 (NT2) cells and ATRA differentiated NTERA2 neurons (NT2N). NT2 cells (A–C) co-express GFAP and SSEA3 with GFAP staining confined to the nucleus (A). NT2 cells also co-express GFAP and β-tubulin III (BT3) which are both mainly confined to the nucleus (B). Nestin with GFAP are co-localized in NT2 cells (C). NT2N assembled themselves into cell clusters which co-expressed GFAP and BT3 found within the cytoplasm and neurites (D). Arrow heads point to cells that appear as radial glia cells which only display GFAP staining (D). NT2N also express neural markers TAU and MAP2 with MAP2 confined to dendrites (tapering morphology shown by arrow heads) and TAU not displaying restriction to axons. In merge panel insert shows close-up view of neurite (E). Synapsin 1 staining is seen as punctate along neurite projections co-expressing BT3 (F). Photomicrographs were obtained at 100X (A–C; scale bar 200 µm) and 200X (D–F; scale bar 100 µm).

In order to assess that the characteristic morphological properties were consistent with their immunocytochemical properties NT2 cells and NT2N underwent fluorescence staining with antibodies specific for the undifferentiated progenitor (SSEA3 and nestin), neuron (β-tubulin III, Tau, MAP2, and synapsin 1), astroglial (GFAP and S-100β), or oligodendrocyte (CNPase) phenotypes. As seen in Fig. 1A–C, NT2 cells expressed both SSEA3 and nestin as previously reported [18]. Staining was not seen with the neuron, astroglial or oligodendrocyte markers (data not shown) with the exception of β-tubulin III (BT3) and GFAP. BT3 and GFAP were co-expressed mainly in the nucleus with light staining of the cytoplasm (Fig. 1B). NT2N displayed no staining for undifferentiated progenitor or oligodendrocyte markers (data not shown) but did display staining for all of the neuron markers and GFAP (Fig. 1D–F). GFAP and BT3 were co-expressed and were confined to the cytoplasm and neurites (Fig. 1D). Also, as seen in Fig. 1D there are a small number of what appears to be radial glial cells which display only GFAP staining and a bipolar morphology (one short and one long unbranched process).

NT2N co-express Tau and MAP2 staining within cell bodies (Fig. 1E). Since the NT2N were grouped in cell clusters, neural extensions from one cell body were unable to be identified. However, MAP2 staining within neural extensions was restricted to only a subset of all neural extensions (which appear to be dendrites based on their tapering morphology) emanating from the clusters whereas Tau staining was present in all neural extensions (Fig. 1E). Synapsin staining was puntate along the neurites with lighter staining seen within the neurites and cytoplasm (Fig. 1F).

**Figure 2:**
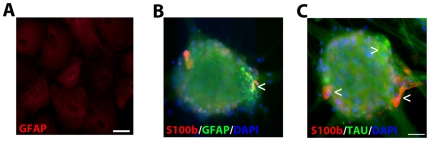
Cell types following all-trans-retinoic acid differentiation of NTera2 cells before purification of neurons. Large protoplasmic astrocytes-like cells that form the basal layer of cells that NTera2 neurons rest on display weak GFAP staining (A). Neural cluster which contains multipolar fibrous astrocytes which co-express GFAP and S-100β (B). The fibrous astrocytes do not display co-expression of S-100β with β-tubulin III positive neurons (C). Photomicrographs were obtained at 100X (A; scale bar 200 µm) and 400X (B–C; scale bar 50 µm).

ATRA differentiated NT2 cells (after mitotic inhibitor treatment but before the purification of NT2N) were stained for GFAP, S-100β, BT3, and Tau (Fig. 2A–C). This approach allowed for the examination of both the neuron and non-neuron phenotypes present in the culture. All cells present in the culture, to some extent, displayed GFAP staining. This included the cell clusters as well as large flat apolar cells appearing morphologically like protoplasmic astrocytes (Fig. 2A) Also, groups of cells, smaller than the large protoplasmic cells but larger than the neural cells, were seen displaying fibroblastic multipolar astrocyte-like morphology. Staining with S-100β revealed that the large protoplasmic cells and most of the cells within the cell clusters did not express S-100β (Fig. 2B). Cells that expressed S-100β were the smaller fibroblastic astroglial-like cells which were also found within the cell clusters. The S-100β cells did express co-localization with GFAP but did not co-localize with Tau indicating the presence of astrocytes (Figure 2B–C).

### NT2N discharge action potentials

**Figure 3:**
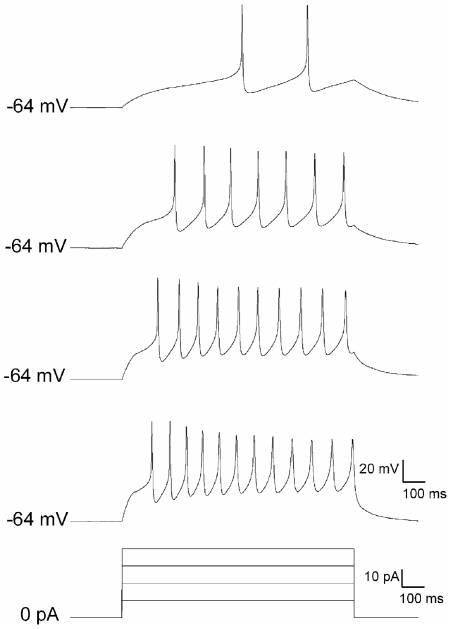
Electrical activity of retinoic acid-differentiated NTERA2 neurons. Representative traces from a cell exhibiting a tonic pattern of action potential discharge in response to direct current injections of 10 pA (top) to 40 pA (10 pA steps; 1 sec duration) from the resting membrane potential.

To confirm that the NT2 neurons possess the intrinsic electrical excitability characteristic of neurons, whole-cell patch clamp recordings were obtained from these cells after 2–3 weeks in neuron purified culture. Under the current-clamp configuration, intracellular current injections were applied from the resting membrane potential, which averaged −61.9±0.4 mV (n = 4). This evoked a tonic pattern of action potential (AP) discharge in all cells examined (Fig. 3), with a mean AP amplitude of 48.5±5.6 mV and a threshold of −26.9±1.3 mV. The minimum current intensity needed to elicit AP discharge (i.e. rheobase) was 27.5±8.5 pA.

To investigate whether the purified neurons establish functional synaptic contacts voltage-clamp recordings were used to examine the prevalence of spontaneous excitatory and inhibitory postsynaptic currents (sEPSCs and sIPSCs, respectively). Despite the presence of an extensive network of neuronal processes, we failed to observe sEPSCs or sIPSCs in any of the sampled cells (n = 10), suggesting that the *in vitro* environment was insufficient to promote synaptogenesis despite clearly promoting neural differentiation.

### “Stemness”, dermal layer, phenotype specific, and growth factor expression

**Figure 4:**
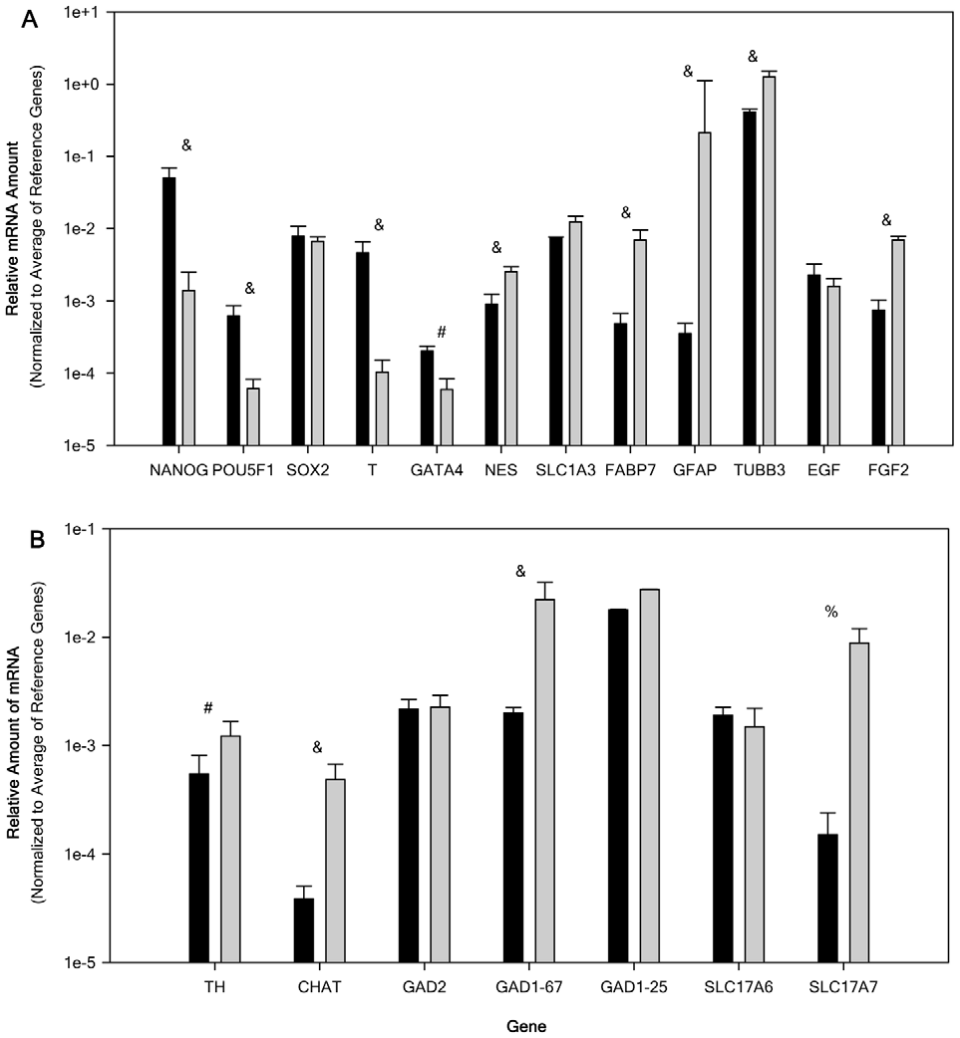
Relative mRNA expression of cell marker genes and neurotransmitter related genes expressed by NTERA2 (NT2) and NTERA2 all-trans retinoic acid differentiated neurons (NT2N). Relative amount of mRNA normalized to the average of 3 reference genes (G3PDH, PPIA, and NUBP1). Relative amount of mRNA for NT2 (black bar) and NT2N (gray bars) were obtained from 6 passage matched cultures (ranging from 25–36) and all samples were analyzed independently in triplicate. Panel A displays the expression of ‘stemness’, dermal layer, cell type makers, and growth factor genes. Panel B displays the relative expression of neurotransmitter related genes. All data are expressed as the average relative gene expression + SEM. For the comparison of NT2 to NT2N: ^#^ p<0.05, ^%^, p<0.01, and ^&^ p<0.005.

The transcriptional regulation of ES cell self-renewal and differentiation involves transcription factors POU class 5 homeobox 1 (POU5F1; Oct3/4), Nanog homeobox (NANOG) and sex determining region Y-box 2 (Sox2) [19]. Down regulation of these genes removes repression of the differentiation genes and allows the ES cells to differentiate. NT2 cells expressed all three genes with NANOG and SOX2 displaying a much higher level of expression compared with POU5F1 (Fig. 4A). Upon differentiation with ATRA, the resulting NT2N expressed a significant down-regulation of NANOG (-71 fold change) and POU5F1 (-8-fold).

Since NT2 cells have been reported to be capable of differentiating into all three dermal layers expression of endodermal (GATA binding protein 4; GATA4), mesodermal (brachyury homolog; T) and ectodermal (nestin; NES) markers were investigated. NT2 cells expressed all 3 dermal markers with the expression of T an order of magnitude greater than NES which was higher than GATA4. NT2N also expressed all 3 genes with only NES being up-regulated (9-fold) and GATA4 and T undergoing down-regulation (-9 and -34-fold respectively).

To confirm the presence of radial glial (RG) cells, we examined the expression of phenotype markers fatty acid binding protein 7 (FABP7) and SLC1A3 (EAAT1/GLAST) which known to be found in this population [20], [21]. Both FABP7 and SLC1A3 mRNA was expressed in the NT2 cells. FABP7 was up-regulated in NT2N (13.7-fold), with no significant change seen in SLC1A3 expression (Fig. 4A), suggesting the maintained presence of or an increase in the RG cell population. In addition, the expression of GFAP and BT3 (TUBB3) were investigated in order validate the previous immunocytochemical findings (Fig. 1). Expression of GFAP was seen in NT2 cells which was up-regulated (207-fold) in NT2N (Fig. 4A), consistent with the increase in GFAP staining seen within this cell population. NT2 cells expressed TUBB3 at a level just below that seen for the average expression of the housekeeping genes which was up-regulated in NT2N (4-fold).

Due to the ability of NT2 cells to proliferate in the absence of feeder cells or the addition of growth factors we examined the expression of fibroblast growth factor 2 (FGF2; β-FGF) and epidermal growth factor (EGF) expression. EGF and FGF2 have been reported to be necessary to maintain pluripotency of hES cells [22]. NT2 cells expressed both EGF and FGF2 which were maintained for EGF and up-regulated for FGF2 (4-fold) in NT2N.

### Neurotransmitter Related Gene Expression

Consistent with the neural morphology, NT2N have been reported to express a variety of neurotransmitter phenotypes [2]. This current investigation focused on the expression of GABAergic, glutamatergic, catecholaminergic, and cholinergic phenotypes. Glutamic acid decarboxylase 65 (GAD65) and glutamic acid decarboxylase 67 (GAD67) are encoded by GAD2 and GAD1-67 respectively. These two enzymes are responsible for the synthesis of γ-aminobutyric acid (GABA). GAD1 contains two alternative splice variants yielding GAD 25 and GAD44 [23]. GAD25 is abundant during early developmental stages but is also present in the adult brain in regions that undergo continuous synaptic rearrangements [23], [24]. This study focused on GAD1-25, GAD2, and GAD1-67 since GAD44 is detected only later in development [23]. All three isoforms were expressed in NT2 cells with GAD1-67 and GAD2 at much lower expression than GAD1-25. NT2N expressed all three isoforms with GAD2 and GAD1-25 displaying no significant change and GAD1-67 significantly up-regulated (9-fold) (see Fig. 4B).

Vesicular glutamate transporter (VGLUT) confers glutamate uptake activity to synaptic vesicles. Three isoforms are present VGLUT 1/2/3 encoded by SLC17A7/A6/A8. VGLUT1 and VGLUT2 are segregated in the brain and expressed in presynaptic terminals revealing functional glutamatergic synapse. VGLUT3 is expressed in presynaptic terminals not classically accepted as glutamatergic [25], [26]. VGLUT1 and 2 were studied here due to their specificity for glutamatergic neurons. NT2 cells expressed both SLC17A6 and SLC17A7 with SLC17A7 displaying a much lower level than SLC17A6 (Fig. 4B). NT2N expressed both isoforms studied here with only SLC17A7 (VGLUT1) displaying a significant increase (74-fold).

Tyrosine hydroxylase (TH) is the enzyme responsible for the synthesis of dopamine and is present in all catecholaminergic neurons. NT2 cells expressed TH which displayed a significant increase (5-fold) in NT2N (Fig. 4B).

Choline acetyltransferase (ChAT) is the rate-limiting enzyme in the synthesis of acetylcholine and the most specific indicator of cholinergic neurons. NT2 cells expressed ChAT which was significantly increased (12-fold) in NT2N (Fig. 4B).

### Region-Specific Regulatory Gene Expression

Determination of the regional-specific regulatory genes expressed by NT2N cells was undertaken to determine if *in vitro* differentiation of NT2 cells by ATRA led to the establishment of multiple regional or specific area neural markers of the CNS. NT2N have been intensively studied but the regional differentiation of these cells has not been investigated. Due to the use of ATRA one would expect a caudalization effect to occur yielding neurons of posterior hindbrain and spinal cord fates [27]. The transcriptional markers used in this study were all expressed in NT2 cells with the exception of NK2 homeobox 2 (NKX2-2). NKN2-2 was not consistently expressed in all passages of NT2 cells and when expressed resulted in a C_t_ of 40 or greater (data not given). Within the NT2N population NKX2-2 could not be detected.

**Figure 5:**
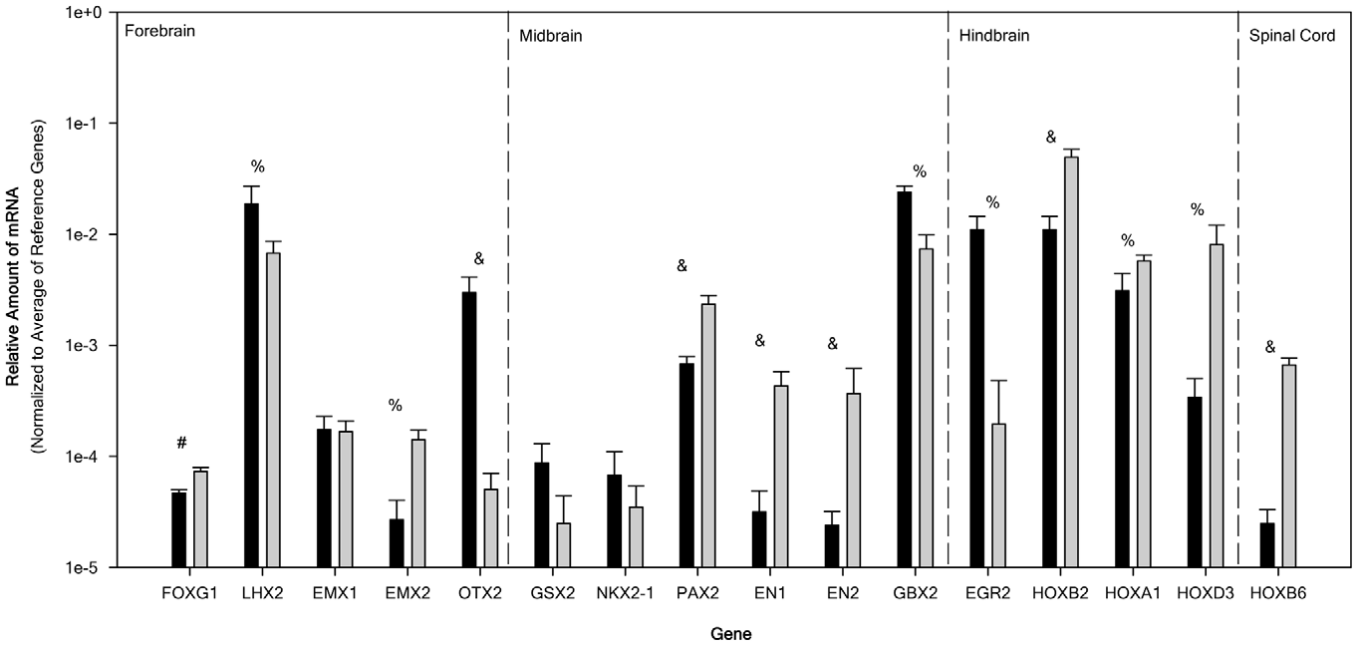
Relative mRNA expression of regional specific transcription factor genes in the rostocaudal direction for NTERA2 (NT2) cells and purified NTERA2 all-trans retinoic acid differentiated neurons (NT2N). Relative amount of mRNA normalized to the average of 3 reference genes (G3PDH, PPIA, and NUBP1). Relative amounts of mRNA for NT2 (black bar) and NT2N (gray bars) were obtained from 6 passage matched cultures (ranging from 25–36) and all samples were analyzed independently in triplicate. Genes are given from rostral to caudal direction (left to right). Dashed lines divide genes for the forebrain, midbrain, hindbrain, and spinal cord. All data are expressed as the average relative gene expression + SEM. For the comparison of NT2 to NT2N: ^#^ p<0.05, ^%^ p<0.01, and ^&^ p<0.005.

Figure 5 shows the relative expression of regional specific transcription factor genes along the rostocaudal axis. Of the rostocaudal markers studied here forebrain makers forkhead box G1 (FOXG1), and empty spiracles homolog 2 (EMX2) were up-regulated (2-fold and 10-fold respectively) with LIM homeobox 2 (LHX2) and forebrain midbrain marker orthodenticle homeobox 2 (OTX2) significantly down-regulated (-3-fold and -67-fold respectively). Of the midbrain-hindbrain markers studied engrailed homeobox 1(EN1), engrailed homeobox 2 (EN2) and paired box 2 (PAX2) were up-regulated (23-fold, 11-fold, and 3-fold respectively) with GS homeobox 2 (GSX2) and NK2 homeobox 1 (NKX2-1) displaying no significant change in their expression. Anterior hindbrain markers gastrulation brain homeobox 2 (GBX2) and rhombomeric maker early growth response 2 (EGR2; Krox20) were down regulated (both by -4-fold) with homeobox (HOX) genes HOXB2, HOXA1, HOXD3, and HOXB6 all displaying up-regulation (6-fold, 3-fold, 10-fold, and 34-fold respectively). The transcriptional factor code expressed by the NT2N population would indicate the presence of progenitors from the forebrain, hindbrain and spinal cord with little presence of midbrain progenitors.

**Figure 6:**
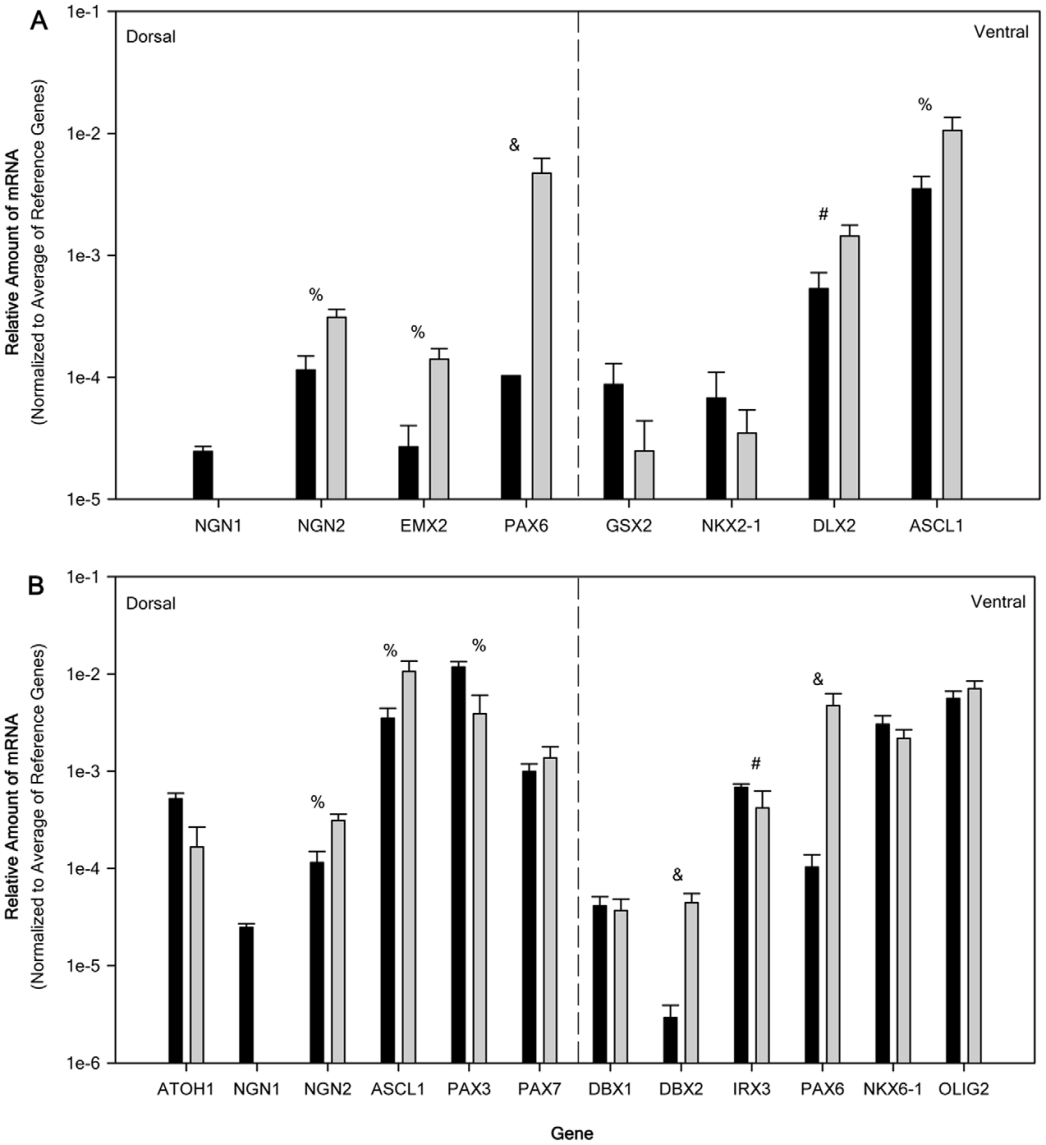
Relative mRNA expression of regional specific transcription factor genes in the dorsoventral direction for NTERA2 (NT2) cells and purified NTERA2 all-trans retinoic acid differentiated neurons (NT2N). Relative amounts of mRNA normalized to the average of 3 reference genes (G3PDH, PPIA, and NUBP1). Relative amount of mRNA for NT2 (black bar) and NT2N (gray bars) were obtained from 6 passage matched cultures (ranging from 25–36) and all samples were analyzed independently in triplicate. Panel A displays the relative expression from the telencephalon and panel B from the spinal cord in a dorsal to ventral order. Dashed line divide dorsal and ventral genes. All data are expressed as the average relative gene expression + SEM. For the comparison of NT2 to NT2N: ^#^ p<0.05, ^%^, p<0.01, and ^&^ p<0.005.

Figure 6 shows the relative expression of regional specific transcription factor genes in the dorsoventral axis for telencephalon-related (Fig. 6A) and spinal cord-related genes (Fig. 6B). It should be noted that some overlap in the two groups exists because genes such as paired box 6 (PAX6) are expressed in multiple CNS regions and the NT2 cells generate a mixed population of neural progenitors.

The dorsal telencephalon transcription factors neurogenin 2 (NGN2), EMX2, and PAX6 as well as ventral transcription factors distal-less homeobox 2 (DLX2) and achaete-scute complex homolog 1 (ASCL1; Mash1) were up-regulated (6-fold, 10-fold, 71-fold, and 3-fold respectively) in the NT2N population (Fig. 6A). Telencephalonic ventral markers GSX2 and NKX2-1 showed no significant change while dorsal marker neurogenin 1 (NGN1) expression could not be detected in NT2N.

The dorsal spinal cord transcription factors NGN2 and ASCL1 were up-regulated (6-fold and 3-fold respectively), while atonal homolog 1 (ATOH1; Math1) and paired box 7 (PAX7) showed no significant change and paired box 3 (PAX3) and NGN1 were down-regulated (-7-fold and not detected respectively). The ventral spinal cord transcription factors developing brain homeobox 2 (DBX2) and paired box 6 (PAX6) were up-regulated (17-fold and 71-fold respectively), developing brain homeobox 1 (DBX1), NK6 homeobox 1 (NKX6-1), and oligodendrocyte lineage transcription factor 2 (OLIG2) showed no significant change, and Iroquois homeobox 3 (IRX3) was down-regulated (-7-fold) within the NT2N population. The transcriptional factor code expressed by the NT2N population represents the presence of both dorsal and ventral progenitors that are found within the telencephalon and spinal cord. The data however, does not imply that all progenitor types found in these two regions of the CNS are present.

## Discussion

While it has been clearly established that NT2 cells can differentiate along the neural ectodermal pathway into postmitotic neurons after exposure to ATRA, our study is the first to attempt to characterize the positional identity expressed by this population of NT2N [8], [28], [29], [30], [31], [32], [33]. The results of this study demonstrate that under monolayer culture conditions NT2 cells differentiate into GABAergic and glutamatergic neurons expressing transcription factors typically observed in the dorsal and ventral forebrain, hindbrain, and spinal cord. Remarkably, NT2N maintain a rostral subpopulation of neurons even after differentiation in the presence of the caudalization influence of RA. Given that culture paradigms are able to alter the neurotransmitter phenotypes expressed by hESC and NT2 cells, we further characterized the resultant neurons [34], [35], [36]. We found that NT2N express neurotransmitter phenotypes that are consistent with previous reports and are electrically active but are immature since they do not form functional synapses. Also, the presence of astrocytes and possible radial glia was noted with no indication of the presence of oligodendrocytes.

### Regional specification of NT2N

Analysis of the expression of regional specific transcription factors along the rostocaudal axis revealed that NT2 cells, although expressing all the rostocaudal genes examined here, did show high expression levels of OTX2 and LHX2 (relative mRNA levels >10^3^) indicating the presence of anterior genes mainly of cortical fates. These cells also highly expressed PAX2, GBX2, EGR2, HOXB2, and HOXA1 which are characteristic of hindbrain fates as well as rhombomeric marker EGR2 (Krox20). More caudal markers HOXD3, and HOXB6 were detected in decreasing amounts again consistent with hindbrain fates. Expression of midbrain markers did not rise to this level of expression. This transcription code would indicate that both forebrain and hindbrain fates are present within the population of NT2 cells, suggesting that NT2 cells express an early neuroepithelial stem cell population similar to that reported for neural rosettes [37]. Since NT2 cells have been reported not to undergo spontaneous differentiation, unlike ES cells, the presence of hindbrain transcription factors may be due to the caudalizing effect of the culture environment as suggested by the high expression of FGF2 seen here [38].

NT2N transcription factor code indicates the presence of forebrain, hindbrain, and spinal cord transcriptional markers. Due to the use of ATRA one would expect a caudalization effect to occur yielding neurons of the posterior hindbrain and spinal cord fates. This is seen with the NT2N population with a movement towards the expression of a more caudal hindbrain fate and the up-regulation of genes of the spinal cord fate. However, this occurs without the loss of cortical fates. The known caudalization effect of RA is somehow resisted by the neural progenitors of this cell population. One possibility is that RA induces the expression of PAX6 [39]. In the E10 mouse, when the neural tube is undergoing regionalization, PAX6 is expressed in forebrain, hindbrain and spinal cord which concur with the neural fates seen in the NT2N population [40]. PAX6 is able in a context-dependent manner to either promote neuroepithelial proliferation or differentiation and its loss results in a 50% reduction of RG and cortical neurons [41]. Therefore, PAX6 may maintain proliferation of a subpopulation of cells which continually provide new neural precursors which maintains the rostral fate seen within the NT2N population. In addition to PAX6, the high expression of NANOG even after differentiation with ATRA may also contribute to the resistance of NT2 cells to either caudalize or undergo differentiation given that the high expression of NANOG suppresses the differentiation of ESCs by RA [42].

Rostocaudal patterning of the neural ectoderm initially occurs in the late blastula through gastrula stages [43] It has been reported that the presumptive rostral neural ectoderm expresses CYP26, from the late blastula onwards, which encodes retinoic acid 4-hydroxylase which specifically degrades RA. The earliest known marker for caudal ectoderm is HOXB1b which is expressed in early gastrula and displays a complimentary pattern to CYP26 [44]. Kudoh et al. have hypothesized that caudalization occurs by suppression of rostral fates in a two-step process [43]. The first step appears to be regulated by FGFs and WNTs which down-regulates CYP26, followed by up-regulation of caudal genes by RA. This would suggest that in order for the rostral population to survive caudalization CYP26 expression needs to be maintained. The NT2 cell population is not homogeneous as evidenced by their expression of rostocaudal genes as presented here. Therefore, a population of rostral progenitors is contained within this population that upon addition of RA is able to maintain itself without being caudalized. We would propose that the rostral population of progenitors would express CYP26. Previous work has shown that there is an antagonism between FGF and RA signaling in controlling neural differentiation [45]. We have shown that FGF2 is expressed in NT2 cells and is further up-regulated in the purified NT2N population which would suggest that other FGFs and possibly other classes of morphogens are expressed endogenously by these cells. Since RA up-regulates the expression of CYP26 and as studied here is added exogenously, in a concentration much higher than physiological, these rostral progenitors should be able to resist FGFs and/or WNTs down-regulation influence on CYP26 and, therefore, resist caudalization [46].

The transcription factors expressed in the forebrain displayed an up-regulation or no change in their expression in the NT2N population with the exception of OTX2 and LHX2. The down-regulation of LHX2 still results in very high level of expression and therefore;, would maintain the expression of cortical fates. The very low level of OTX2 expression seen here could indicate a movement away from the rostral fate. OTX2 is widely expressed throughout the forebrain and midbrain where it defines areas of the rostral brain of E8-E10 mouse embryos. However, starting from E10.75 OTX2 expression in the murine cerebral cortex rapidly disappears just prior to the onset of neurogenesis at E11.0 [47]. This would suggest that the loss in the expression of OTX2 seen in the NT2N population is not necessarily a movement to a more caudal fate but just the normal loss of OTX2 during neurogenesis.

In the dorsoventral axis the expression of regional transcription factors needs to be considered in respect to the rostocaudal region. As a result the dorsoventral transcription factors were evaluated separately for the telencephalon and the spinal cord. NT2 cells express all telencephalonic transcription factors studied here with high expression being seen in ventral (subpallial) factors DLX2 and ASCL1. DLX2 and ASCL1 are expressed in both the lateral and medial ganglionic eminences (LGE and MGE) *in vivo* which give rise to GABAergic cortical interneurons and cholinergic telencephalic neurons [48]. DLX2 has been show to induce GAD1-67 [49]. However, within the MGE, NKX2-1 controls the development of both telencephalic cholinergic neurons and GABAergic cortical interneurons and its removal results in a loss of 50% of the GABAergic interneurons and a total loss of cholinergic neurons [48]. The weak expression of NKX2-1 in NT2 cells would thus predict a reduced development of cholinergic neurons which is consistent with the high expression of GAD and the very low expression of CHAT seen in the NT2 cell population.

The NT2N population displays an up-regulation of DLX2 and ASCL1 and no change in NKX2.1 expression. This suggests that the same ventral fate seen in NT2 cells is present within the NT2N population. Up-regulation of the genes responsible for the dorsal telencephalon progenitors is seen following ATRA differentiation. However, the expression of NGN1 is undetectable. NGN2 is a downstream target for PAX6 and its up-regulation is most likely a direct result of PAX6 expression [50]. In NT2 cells RA has been reported to up-regulate NGN1 throughout the 21 days of the ATRA differentiation [30]. It appears that the loss of NGN1 expression may be due to the removal of ATRA or that the use of the mitotic inhibitors may have removed the population of NGN1 expressing progenitors. Up-regulation of NGN2, EMX2, and PAX6 define the dorsal telencephalon by inhibiting expression of ventral markers. This leads to the development of glutamatergic projection neurons (pyramidal). Pyramidal neurons preferentially express SLC17A7 (VGLUT1) [51]. The development of pyramidal neurons is consistent with the up-regulation of SLC17A7 and no change in the expression of SLC17A6 seen in the NT2N population.

NT2 cells express all of the spinal cord dorsoventral axis transcription factors studied here with high expression of ASCL1, PAX3, PAX7, NKX6.1, and OLIG2 indicating the present of both dorsal and ventral neural progenitors. Given the expression of transcription factors expressed in NT2 cells the intermediate progenitor domains of ventral progenitor 0 (vp0), and vp1 and the most ventral domain vp3 are poorly expressed or lacking.

Following exposure of NT2 cells to ATRA the NT2N population expresses nearly the same progenitor populations as expressed in NT2 cells. NKX2.2 expression is totally absent following ATRA and would suggest the absence of vp3 progenitors. The expression of OLIG2 would suggest the possible development of motor neurons which requires the association of OLIG2 with NGN2. However, the development of oligodendrocytes requires the interaction of OLIG2 with NKX2-2. Since NKX2-2 is undetectable in the NT2N population the lack of oligodendrocyte development seen in this population is consistent with the loss of NKX2.2 expression [52], [53]. An increase in the progenitor population from vp0, and vp1 is seen with the NT2N population due to the up-regulation of DBX2 which is consistent with the reported dependence of these progenitor regions on RA for their development [54].

### Neuronal characterization of NT2 cells and NT2N

NT2N described here appear to be immature due to the lack of restriction of TAU to axons and their lack of formation of functional synapses. The punctate staining seen with Synapsin 1 has been previously reported to occur in NT2N cultured with or without astrocytes [12], [29]. Here we report that the NT2N possess intrinsic electrical excitability characteristic of neurons but fail to establish functional synaptic contacts despite their extensive network of neural processes. This result appears to be in agreement with previous reports that the NT2N as well as other neurons generated from hES cell require cell contact with mature astrocytes in order to induce synaptogenesis [12], [55].

The presence of different neurotransmitter phenotypes has been previously reported for both monolayer [2] and aggregate differentiation methods [29]. In agreement with previous reports, the population of NT2N characterized here displayed up-regulation of genes directly linked to the GABAergic, glutamatergic, catecholaminergic, and cholinergic phenotypes.

We also report the presence of astrocytes in the ATRA-differentiated cell population before isolation of NT2N and within the cell clusters present in the purified NT2N population. The generation of oligodendrocytes, was not seen. This may be due to the inability of NT2 cells to differentiate into oligodendrocytes, as supported by the lack of NKX2-2 expression, and is agreement with other studies using human neurospheres or hES cell lines [56], [57].

As expected, significant down-regulation of the “stemness” genes NANOG and POU5F1 was observed although SOX2 expression was maintained. This finding is in agreement with previous work [58]. Upon examination of dermal markers expressed by NT2 cells endodermal, mesodermal, and ectodermal markers were all present. However, only the ectodermal marker was up-regulated in response to ATRA triggered differentiation while both endodermal and mesodermal markers were down-regulated. This result is consistent with the maintained SOX2 levels forcing the differentiation of ES cells along the ectodermal differentiation pathway at the expense of endodermal and mesodermal cell fate [58].

Immunocytochemical analysis of the NT2 and NT2N cell populations did display some differences from what has been previously reported for these cells [29], [32], [57], [59]. The NT2 and NT2N cells displayed co-localized staining for both GFAP and BT3 which was confirmed by the expression of both BT3 (TUBB3) and GFAP (GFAP) mRNA. GFAP staining of BT3^+^ neurons has been previously reported *in vitro* and was attributed to the presence of serum-containing medium [60]. The presence of serum used here to maintain NT2N could account for the co-localization of BT3 and GFAP. BT3 staining of undifferentiated NT2 cells is consistent with the reported BT3 staining of undifferentiated hES cell lines [57]. The expression of GFAP in NT2 cells is surprising but may be accounted for by the fact that these cells were derived from an adult in contrast to hES cells derivation from the blastocyst. Human adult neural stem cells within the subventricular zone display GFAP [61]. EC cells are derived from carcinoma in situ (CIS) cells showing high similarity to primordial germ cell/gonocytes, which through a process of adaptation undergo dedifferentiation to become EC cells [62], [63]. Since NT2 cells are EC cells which are derived from a restricted embryonic/neonatal precursor cell developed in an adult niche one might hypothesize that expression of GFAP in theses adult-derived cancerous stem cells may occur. However, in the human midgestational fetus *in vivo* co-expression of GFAP, BT3, and nestin is seen in the telencephalic VZ/SVZ at the ganglionic eminence and the developing cortical plate [64]. This correlates with the expression of these three genes at both the protein and mRNA levels in NT2 cells and may indicate that NT2 cells express an early neural progenitor phenotype as previously reported by Pleasure et al [59]. This correlates with the expression of these three genes at both the protein and mRNA levels in NT2 cells. This expression pattern combined with the expression of FABP7 and SLC1A3 are consistent with human RG or neural precursor cells [21]. Based on their bipolar morphology RG cells appear to be present within the cell clusters of the NT2N population which is consistent with the differentiation pathway seen during both *in vivo* and *in vitro* differentiation of hESC [35], [65], [66], [67].

Unlike hES cells, NT2 cells do not require either the use of feeder cells or the addition of FGF2 and EGF. The high expression level of NANOG may account for the ability of NT2 cells to grow well in the absence of feeder cells as has been reported for hES cells expressing high levels of NANOG [68]. Furthermore, human EC cells have been reported to produce FGF2 [69], [70]. We have confirmed that NT2 cells express FGF2 as well as EGF mRNA. If the expression of mRNA for both FGF2 and EGF translate into protein expression, as reported for FGF2, this may also account for the ability of NT2 cells to self-replicate without the need for feeder cells or additional growth factors.

### Conclusion

Given the transcription factor code expressed by NT2 cells and NT2N it appears that NT2 cells under the paradigm studied here are restricted to areas of PAX6 expression, namely the forebrain, hindbrain, and spinal cord, yielding both dorsal and ventral progenitors from these areas. This restricted transcriptional code resulted in both glutamatergic and GABAergic neurotransmitter phenotypes from these regions which contrasts with the preferential GABAergic phenotype seen in long-term expanded primary and ES cell derived neural precursors [71], [72]. The maintained expression of a rostral precursor population in the presence of both endogenous and exogenous caudalization factors is a surprise and the relevant underlying mechanism remains unclear. It can be hypothesized that the total NT2 cell population does not undergo differentiation at the same rate and even under the influences of growth factors and/or morphogens a population of early neural progenitors is maintained possibly through the high expression of PAX6 and/or NANOG. This may be a condition of the EC cell malignant state. Yet this cell line provides both a model of human neural cell differentiation and a source of a diverse population of human central nervous system neurons which are amenable to further study. Given that NT2N cells have been used for phase 1 and 2 clinical trials in stable stroke patients and to date (10 and 5 years after publication of the trials respectively) transplanted NT2N have demonstrated consistent non-tumorigenic outcomes and are as efficacious as human fetal cells, these neurons may be cautiously viewed as useful for therapeutic use [15]. Understanding the regional specification of the neurons derived from pluripotent stem cells may facilitate their integration and aid the design of effective strategies for neural transplantation within the CNS.

## Materials and Methods

### Cell Culture

NTERA-2 cl.D1 (NT2) cells were obtained from American Type Culture Collection (CRL-1973; Manassas, VA) and maintained in Dulbecco's modified Eagle's medium (high glucose) containing 10% fetal bovine serum (FBS), 2 mM L-alanyl-L-glutamine (AG), 25 mM HEPES buffer (pH7.3), 1X minimum essential medium non-essential amino acids, 56 µM β-mercaptoethanol (β-ME), and 25 µg/ml gentamicin sulfate (GS). The cells were fed twice a week and split 1∶4 when confluent by mechanical scraping. For differentiation with retinoic acid NT2 cells were trypsinized with 0.05% trypsin/EDTA and plated at 2.7×10^4^cells/cm^2^ in Opti-MEM I (Invitrogen; Carlsbad, CA) containing 4% FBS, 2 mM AG, 56 µM βME, 25 µg/ml GS, and 10 µM all-trans retinoic acid (ATRA; Sigma-Aldrich, St. Louis). Cell were fed twice a week for four weeks and the neurons were isolated in accordance with Pleasure et al. [8]. Briefly, following ATRA differentiation, cells were replated at 1∶6. After 1–2 days cultures were mechanically shaken to dislodge cells and these free-floating cells were plated onto poly-L-ornithine (PLO)/Matrigel or Geltrex coated growth surface in Opti-MEM I containing 4% FBS, 2 mM AG, 56 µM βME, 25 µg/ml GS, 1 mM cytosine arabinoside, 10 mM fluorodexoyuridine, and 10 mM uridine. Cells were fed twice a week for four weeks. Following this treatment, purified neurons were isolated by mechanically dislodging the neurons from the basal layer of cells and replated on PLO/Matrigel or Geltrex in the same media without cytosine arabinoside, fluorodexoyuridine, or uridine.

### Antibodies and Immunofluorescence

For immunofluorescence NT2 cells or purified ATRA differentiated NT2 cell derived neurons (NT2/N) were grown on 12 mm glass coverslips coated with poly-L-ornithine (PLO; Sigma)/Matragel (Becton Dickerson) and washed with phosphate buffered saline (PBS, pH 7.2). The cells were then fixed for 20 min in 4% paraformaldehyde and 4% sucrose in PBS. The cells were then washed 2X with PBS and permeabilized for 20 min with 0.3% Triton X-100 in PBS. The cells were then washed with PBS and blocked for 1 hr with 1% bovine serum albumin in PBS. The cells were then incubated for 2 hr with the primary antibody at the listed dilutions in PBS. Following primary antibody incubation the cells were washed 3X with PBS and incubated with the appropriate secondary antibody for 1 hr in PBS. Following washing 3X in PBS the coverslips were mounted for microscopy in Vectashield hard-set mounting medium (Vector Laboratories, Burlingame, CA). Images were acquired using a Nikon Microphot-SA microscope with a SensiCam cooled CCD camera (Cooke Corp., Eugene, OR) and using CamWare image acquisition software (Ver 2.13, Cooke Corp.).

Anti-human stage specific embryonic antigen 3 (SSEA3) (sc-21703, Santa Cruz Biotechnology, CA), anti-tubulin, beta III isoform (MAB1637, Chemicon International), and anti-2′,3′-cyclic nucleotide 3′-phosphodiesterase (CNPase) monoclonal antibodies (C 5922, Sigma-Aldrich) were used at a dilution of 1∶50. Anti-nestin monoclonal human specific antibody (MAB5326, Millipore), anti-microtubules associated protein 2 (MAP2) monoclonal antibody (13–1500, Invitrogen), anti-TAU polyclonal antibody (AHB0351, Invitrogen), anti-glial fibrillary acidic protein (GFAP) IgG purified polyclonal antibody (G 9269, Sigma-Aldrich), and. anti-synapsin 1 polyclonal antibody (A6442, Invitrogen) were used at a dilution of 1∶200. Fluorescein (FITC) conjugated goat anti-mouse IgG (115-095-062), FITC-conjugated goat anti-rat IgM (112-095-075), FITC-conjugated goat anti-rabbit IgG (111-095-144), Cy3-conjugate goat anti mouse (115-095-146) and Cy3-conjugated goat anti-rabbit (111-165-144) (Jackson ImmunoResearch, West Grove, PA) were used at a dilution of 1∶100.

### Electrophysiological recordings

Coverslips with NT2N were transferred to a submersion-type chamber (RC-22; Warner Instruments, Hamden, CT), mounted on the stage of an upright microscope (BX51WI; Olympus, Center Valley, PA) and perfused at room temperature with oxygenated artificial CSF (aCSF) solution containing the following (in mM): 125 NaCl, 2.5 KCl, 25 NaHCO_3_, 1.0 NaH_2_PO_4_, 1.0 MgCl_2_, 2.0 CaCl_2_, and 25 glucose, at a rate of 1.5–3 ml/min.

Recording electrodes were constructed from thin-walled single-filamented borosilicate glass (1.5 mm outer diameter; World Precision Instruments, Sarasota, FL) using a microelectrode puller (Sutter Instruments, Novato, CA). Pipette resistances ranged from 5 to 7 MΩ and seal resistances were >1 GΩ. For voltage-clamp experiments, patch electrodes were filled with a solution containing the following (in mM): 130 Cs-gluconate, 10 CsCl, 10 HEPES, 11 EGTA, 1.0 CaCl_2_, and 2.0 MgATP, pH 7.2 (300–305 mOsm). EPSCs were isolated at a holding potential (hp) of −70 mV while IPSCs were recorded at a hp of 0 mV, thus minimizing the contribution of NMDA and AMPA/kainate receptor-mediated events [73]. Current clamp experiments used an electrode solution of (mM): 130 potassium gluconate, 10 KCl, 10 Hepes, 1.0 EGTA, 0.1 CaCl_2_, 2.0 MgATP, pH 7.2 (300–305 mOsm). Neurons were visualized with infrared-differential interference contrast and whole-cell patch-clamp recordings were obtained using a Multiclamp 700B amplifier (Molecular Devices, Sunnyvale, CA). Under the current-clamp configuration, action potentials were evoked via current injection through the recording electrode (10–50 pA in 10 pA steps; 1 sec duration).

Membrane voltages were adjusted for liquid junction potentials (approximately –14 mV) calculated using JPCalc software (P. Barry, University of New South Wales, Sydney, Australia; modified for Molecular Devices). Currents were filtered at 4–6 kHz through a –3 dB, four-pole low-pass Bessel filter, digitally sampled at 20 kHz, and stored on a personal computer (ICT, Cincinnati, OH) using a commercially available data acquisition system (Digidata 1440A with pClamp 10.0 software; Molecular Devices). All data are expressed as mean ± SEM.

### RNA Isolation and cDNA Preparation

Total RNA was collected from passage matched (ranging from 25–36) NT2 and NT2N cultures (N = 6/experiment) following a HBSS w/o wash. RNA extraction and DNase I treatment were accomplished using the Absolutely RNA Purification Kit according to the manufacturer's instructions (Stratagene, La Jolla, CA). The RNA was quantitated using a fluorescence method (Quant-iT RNA assay Kit and Qubit Fluorometer; Invitrogen). The isolated RNA was aliquoted and stored at −75°C in 10 mM Tris-HCl buffer (pH 8.0) until use. One µg of total RNA per reaction, 4 reactions per sample, were reverse transcribed in a volume of 20 µl, using the iScript cDNA Synthesis Kit (Bio-Rad Laboratories, Hercules, CA) by incubation at 25°C for 10 min, 42°C for 45 min, 85°C for 5 min followed by a hold at 4°C. The first strand cDNA reaction was stored at −20°C until use.

### Oligonucleotide Primers

Primers were designed using Primer-BLAST software which incorporates Primer3 software to design the primers and a BLAST search of the primers against a user selected database (Homo sapiens Refseq RNA database). (www.ncbi.nlm.nih.gov/tools/primer-blast/) [74]. All primers were designed to allow for specific amplification of the gene specific mRNA independent of splice variants (when possible), to anneal at 60°C, and to traverse an intron boundary (see Supporting Information Table S1). All amplicons were confirmed by agarose electrophoresis to assess if the amplicon was the predicted size and a single product. All oligonucleotides used in this study were synthesized by Operon Biotechnologies Inc. (Huntsville, AL).

### Quantitative RT-PCR

Quantitative RT-PCR (qPCR) was performed on the MPx3005 instrument (Stratagene). Each 25 µl reaction included 12.5 µl of DyNAmo HS SYBR Green 2X Master mix (New England Biolabs, Inc.; Ipswich, MA) containing 2.5 µM MgCl_2_, 0.3 µM each forward and reverse primer, 2 µM ROX reference dye, and 25 ng of cDNA. The experiment was performed following the following protocol: activation of the Taq polymerase at 95°C for 15 min, followed by 45 cycles of denaturing at 95°C for 15 sec, annealing at 60°C for 1 min, extension at 72°C for 1 min followed by fluorescence measurement at 516 and 610 nm (SYBER Green and ROX respectively). This was followed by a melting curve analysis. All experiments included no-template controls and all samples were analyzed independently in triplicate. The determination of PCR efficiency, threshold cycle (C_t_) determination, and RNA starting concentration were calculated using raw fluorescence data using LinRegPCR analysis software [75], [76].

### Statistical Analysis

Groupwise comparison of gene expression ratios was performed by REST-2009 (December 2009 release) [77]. Expression data was normalized to the geometric mean of three housekeeping genes: glyceraldehyde-3-phosphate dehydrogenase (G3PDH), peptidylprolyl isomerase A (cyclophilin A; PPIA), and nucleotide binding protein 1 (NUBP1). Randomization was conducted using 6,000 permutations for statistical evaluation. The REST 2009 program incorporates correction for amplification efficiencies (as determined by LinReg) into the calculation of gene expression ratios. The level of significance was determined as p<0.05.

## Supporting Information

Table S1 **Primer sequences and amplicon size.**
(DOCX)
