# Supplementary material for: Regional Differentiation of Retinoic Acid-Induced Human Pluripotent Embryonic Carcinoma Stem Cell Neurons
Source: PLoS One. 2011 Jan 20;6(1):e16174. doi: 10.1371/journal.pone.0016174 (PMC3024414; doi:10.1371/journal.pone.0016174)
Supplement: Table S1 — Primer sequences and amplicon size. (DOCX) [file pone.0016174.s001.tif]

**Table S1.** **Primer sequences and amplicon size.**

| **Gene Symbol** | **Protein** | **Forward Primer** | **Reverse Primer** | **Primer Bank ID** | **Size** |
| --- | --- | --- | --- | --- | --- |
|  |  |  |  |  |  |
| **Housekeeping** | |  |  |  |  |
| PPIA | Cyclophlin A | cagacaaggtcccaaagacag | ttgccatccaaccactcagtc | 10863927a1 | 298 |
| NUBP1 |  | tggcctagcagaggatgaaaa | gtcttccacgtacactggaga | 4505337a2 | 142 |
| G3PDH |  | catgagaagtatgacaacagcct | agtccttccacgataccaaagt | 7669492a3 | 113 |
|  |  |  |  |  |  |
| **Stemness** |  |  |  |  |  |
| NANOG |  | caaaggcaaacaacccactt | tctgctggaggctgaggtat | Primer BLAST | 158 |
| SOX2 |  | gccgagtggaaacttttgtcg | gcagcgtgtacttatccttctt | 28195386a1 | 154 |
| POU5F1 | OCT4 | cttgaatcccgaatggaaaggg | gtgtatatcccagggtgatcctc | 4505967a1 | 164 |
|  |  |  |  |  |  |
| **Neurotransmitter** | |  |  |  |  |
| CHAT |  | gtcccccgtaagatggcag | cacccagttggctgtcttct | 10181098a1 | 240 |
| TH |  | gagacgtttgaagccaaaatcc | aggtcagggtcgaacttggt | 4507481a1 | 251 |
| SLC17A6 | VGLUT2 | tcatcactcagattccgggag | cacaccctcaacaagtccctg | 9966811a3 | 173 |
| SLC17A7 | VGLUT1 | ttttctggggctacattgtcac | actccgttctaagggtgggg | 9945322a1 | 245 |
| GAD2 | GAD65 | ggcttttggtctttcgggtc | gcacagtttgtttccgatgcc | 4503875a1 | 117 |
| GAD1 GAD67 | GAD67 | catgaagatccttccactgaaca | ggttccgttgtccaccaatc | 4557603a3 | 139 |
| GAD1 GAD25 | GAD25 | gcggaccccaataccactaac | acaaggcgactcttctcttcc | 15451889a3 | 143 |
|  |  |  |  |  |  |
| **Cell-Type Markers** | |  |  |  |  |
| TUBB3 | β-tubulin III | cggtggtggaaccctacaac | aggtggtgactccgctcat | 5174737a1 | 183 |
| GFAP |  | cctctccctggctcgaatg | ggaagcgaaccttctcgatgta | 4503979a1 | 161 |
| FABP7 |  | acatgaaggctctaggcgtg | aagtttgtctccatccaggc | Primer BLAST | 218 |
| NES | nestin | aggtagaggagctggcaagg | cacagccagctggaactttt | Primer BLAST | 272 |
| GATA4 |  | cccggcttacatggccgacg | acccgtcccatctcgcctcc | Primer BLAST | 217 |
| T | brachyury | cctctccctcccctccacgc | ggtgggctggcattgtggct | Primer BLAST | 222 |
| SLC1A3 | EAAT1/GLAST | acaaaggaaaacatgcacagaga | gcaggcttctaccagatttgg | 31543628a3 | 108 |
|  |  |  |  |  |  |
| **Growth Factors** | |  |  |  |  |
| EGF |  | tcgtggtggctgtctgcgtg | gcctggccatcctcaccagc | Primer BLAST | 262 |
| FGF2 |  | gaaaaggcaagatgcaggag | aatgctgaaagagaaggcca | Primer BLAST | 183 |
|  |  |  |  |  |  |
| **Region-Specific Genes** | |  |  |  |  |
| FOXG1 |  | ttgctacatgacttgccagc | tcctatctcccatgtccagc | Primer BLAST | 204 |
| LHX2 |  | ttctaatgactcgcaacccc | agttgttcctcggtccacac | Primer BLAST | 193 |
| EMX1 |  | agagcctggggtggtagatt | ggcccttccctatgtctagc | Primer BLAST | 200 |
| EMX2 |  | taactccagccccataaatccg | ccaggggtagaaggtggac | Primer BLAST | 200 |
| OXT2 |  | agaggacgacgttcactcg | ggcacttagctcttcgattctt | 11119420a3 | 159 |
| GSX2 |  | gggggctataaaatacccga | acatagaaggagcgcgacat | Primer BLAST | 237 |
| NKX2-1 |  | cgcatccaatctcaaggaat | cagagtgtgcccagagtgaa | Primer BLAST | 175 |
| PAX2 |  | tatgttcgcctgggagcttc | gaaaggctgctgaactttgg | Primer BLAST | 118 |
| EN1 |  | ccgcaccaccaactttttcat | tggacagggtctctacctgc | 7710119a1 | 171 |
| EN2 |  | ccggcgtgggtctactgta | ggccgcttgtcctctttgtt | 7710121a1 | 101 |
| GBX2 |  | aatgccaattccaagacagg | agctgggctgtgactttgtt | Primer BLAST | 230 |
| EGR2 | Krox-20 | ggagacctctaccaggaccc | ctgggatcattgggaagaga | Primer BLAST | 139 |
| HOXB2 |  | ctcctgtctccagctatccg | ttttccagtagacggccaag | Primer BLAST | 191 |
| HOXA1 |  | gggtgtcctactcccactca | ggaccatgggagatgagaga | Primer BLAST | 162 |
| HOXD3 |  | agagtctcgacagaactccaag | gcgttccgtgagattcagc | 19923391a2 | 214 |
| HOXB6 |  | gagtgaaggtgctggaaagg | gtgagtccgctcctcagtttc | Primer BLAST | 295 |
| NGN1 |  | gaccctcttttctccttccc | ctttaaagctcccgcttcct | Primer BLAST | 192 |
| NGN2 |  | gagctgccatttctgctacc | gcccgtctgaatgaaggata | Primer BLAST | 196 |
| PAX6 |  | aggtattacgagactggctcc | tcccgcttatactgggctattt | 4505615a2 | 104 |
| DLX2 |  | gcctgaaattcggatagtgaacg | aggggatctcaccacttttcc | 4758168a3 | 236 |
| ASCL1 | Mash1 | acatggctttcagaaaacgg | gctgtgcgtgttagaggtga | Primer BLAST | 191 |
| ATOH1 | Math1 | tgaaggagttgggagaccac | atgtagcaaatactgggcgg | Primer BLAST | 212 |
| PAX3 |  | agccgcatcctgagaagtaa | ttctgcgctgtttcctcttt | Primer BLAST | 202 |
| PAX7 |  | cactgtgaccgaagcactgt | gtcaggttccgactccacat | Primer BLAST | 207 |
| DBX1 |  | cttcctggtggaggatctga | gatggcgttcactccaaact | Primer BLAST | 232 |
| DBX2 |  | aaacttgccatcaacttggg | ttgaaggacatggagaaggg | Primer BLAST | 184 |
| IRX3 |  | tgaaaactagaggagggcga | caggtcaggtccgaacagat | Primer BLAST | 203 |
| NKX6-1 |  | attcgttggggatgacagag | tgggatccagaggcttattg | Primer BLAST | 186 |
| OLIG2 |  | ggacaagctaggaggcagtg | atggcgatgttgaggtcgtg | 7341207a2 | 183 |
| NKX2-2 |  | ttacagaatgtttgcgcagc | agacggctgacaatatcgct | Primer BLAST | 167 |
